# Notes and News

**Published:** 1888-08-25

**Authors:** 


### Notes and News.

Collected and Communicated.

In spite of the bad weather, about 50,000 people attended the Leeds Hospital "Gala" at Roundhay Park, on Monday week.

The British Association and the Pharmaceutical Society have both chosen Bath for the place of their annual assembly this autumn.

Of the 115 patients who have passed through the Dalrymple Home for Habitual Drunkards at Rickmansworth, at least 52 (these have been traced) are doing well.

The Durham Rovers' Football Club, having qualified by an annual subscription of £25 to the hospital, is about to select one of its members as a life governor.

More than three hundred sick children, collected from the poorest districts of London, have been sent into the country for two weeks by the aid of Miss Edith Woodworth's Buttercup and Daisy Fund.

The Mayor of Lowestoft, Mr. W. H. Clubbe, opened a bazaar on the Pier on Wednesday, in aid of the funds of the West Hospital. The bazaar continued open on Wednesday and Thursday, and the weather being fine, drew together large numbers of people.

Mr. S. Montagu, M.P., of Old Broad Street, is a patron of a garden party to be given at the Essex County Cricket Ground, on Wednesday, 29th inst., in aid of the funds of the Shadwell Hospital for Children, Victoria Park Hospital, and the East London General Pension Society.

An appeal on behalf of the Children's Country Holidays Fund is signed by the chairman (the Rev. J. R. Diggle), and several members of the School Board for London. Ten thousand children have already been sent into the country this year through the agency of the fund.

"Amongst the correspondence handed over to us by Mr. Edge" (concerning the Blackburn Infirmary), says the *Blackburn Standard*, "there is a letter from a now remarkable man, Mr. John Morley, addressed Lytham, March 10th, 1862, in which the writer notes his departure from Blackburn for good, and forwards his mite to the relief fund, in a letter as remarkable for its modesty as for its courtesy."

Mr. W. H. Kesteven, writing to the *Times* charges against those hospitals which receive paying patients, that they are "simply prostituting their noble reputation for the sake of the money necessary to maintain the secretaries and other energetic individuals who have been instrumental in getting them built, recognizing in their existence a comfortable source of income for themselves. What have "secretaries and other energetic individuals" to say to this?

A stranger visiting the wards of the Torbay Hospital last Thursday would have been struck with the intense silence, particularly in the women's wards, where there was not one patient. The silence was satisfactorily accounted for. All the nurses and patients were (with the exception of four men) enjoying afternoon tea on Paignton Sands. At two o'clock a very merry party started in two large breaks, to the number of fifty. A good tea and a lovely warm afternoon were appreciated, and at half-past seven all returned, much the better for their outing.

About forty years ago Joseph Wiggins, a young sailor, conceived the idea that it was possible at certain periods to navigate the Kara Sea, and to open out Siberia to British enterprise. A little more than twenty years ago, at his own cost, he equipped a vessel and proved his theory true. Today Kara is navigated by British vessels, and Siberia has become a market for British commerce. Such is the stuff that British sailors are made of! What is to be done for Joseph Wiggins?

A coroner's jury at the London Hospital have again emphasized the fact that young children in small houses are constantly in danger of being burned to death. A mother, in the room with her two children, had occasion to go into her back garden, leaving the elder, aged five, to take care of the younger, a mere infant. There was no fire screen or other protection. In a few moments screams were heard from the house, and the mother rushed back to find the infant in flames. Of course the verdict was one of accidental death. But it is shocking to think of two little children, one of them a baby, and the other little more, left alone together in a room with an open fireplace. When will mothers learn that the saving of a few shillings—the cost of a fireguard—exposes their children almost every hour in the day to the danger of an agonizing death?

The following vacancies are announced this week, the amount of salary in each case being given after the name of the institution :—

**Resident Medical Officers.**

Belgrave Hospital for Children. House Surgeon and Assistant Secretary.—£30 per annum, with board, etc.
Borough of Sheffield, Lodge Moor Hospital for Infectious Diseases. Resident Medical Officer.—£150 per annum, with board, etc.
Borough of Sheffield, Winter Street Hospital for Infectious Diseases. Resident Medical Officer.—£200 per annum, with board, etc.
City of Liverpool Infectious Diseases Hospital. Resident Medical Officer.—£100 per annum, with board, etc.
General Hospital, Birmingham. Assistant House Surgeon.—Board; no salary.
General Hospital, Nottingham. Junior Resident Medical Officer. —£100 for first year, with increase of £10 per annum to £120.
Liverpool Dispensaries. Assistant Surgeon.—£80 per annum, with board, etc.
Liverpool Northern Hospital. Assistant House Surgeon.—£70 per annum, with board, etc.
Royal Berks Hospital, Reading. Assistant House Surgeon.—Board; no salary.
Royal Hospital of Bethlem. Assistant Medical Officer.—£200 per annum, residence.
St. Mark's Hospital for Fistula, &c., City Road, E.C. House Surgeon.—£50 per annum, with board, etc.
Victoria Hospital, Burnley. Resident Medical Officer.—£80.

**Non-resident Medical Officers.**

King's County Infirmary, Tullamore. Surgeon.—£94 per annum.
Medical Missionaries for Magila, S. Africa and Madagascar. Apply to R. H. Combes, Secretary, Guild of St. Luke, Galeston, Eaton Avenue, Hampstead, N.W.
Parish of Ochiltree. Medical Officer.—£20 per annum.
Swinford Union, Low Park Dispensary. Medical Officer.—£120 per annum, and fees.
University of Aberdeen. Chair of Chemistry.
Winchcombe Union. Medical Officer.—£65 per annum, and fees.

---

In anticipation of the projected "Women's Congress," to be held next year, a managing committee, it is stated, has been appointed by the "Society for the Amelioration of the Lot of Women, and the Claiming of their Rights," acting conjointly with the "Woman's Federation" and the "League for the Protection of Women." The proceedings are to extend over a month, and 25,000 representative women are expected to take part. It is not stated whether the whole 25,000 are to speak together, or in turn. Husbands and other males are to be excluded.

---

The Hastings newspapers are full of praise of working-men in connexion with their generous giving on Hospital Saturday. One local organ points out that the Saturday collection is within £50 of the amount collected in London on the first Hospital Saturday. It is almost half as large as the Hastings Sunday collection. The husband of one of the collectors says: "It was wonderful how the working-men gave. They dropped even their sixpences into my wife's box; and fishermen trundling their barrows on the road came over with their contributions." It seems plain that working men are becoming fully alive to their duties and responsibilities in regard to hospitals.

---

On Saturday, the 14th inst., the committee of the Bradford Workpeople's Ambulance presented to the Infirmary of Bradford a two-wheeled Ashford's ambulance litter, which is the first result of the endeavours of that committee to provide improved means of conveyance to hospital for persons who are so unfortunate as to meet with accident. The litter is complete, and possesses all the latest patents and improvements, including telescopic handles. The committee are also having made a horse ambulance carriage, and will, in the course of a few weeks, present this, through the Corporation, to the town, for the use and benefit of its inhabitants. The carriage is so constructed that by changing the shafts it can be run with either one or two horses. It will hold two patients, and also provide sufficient room for a doctor or an attendant.

The Earl of Clarendon presided at the annual meeting of the Watford Cottage Hospital on Thursday. The Salters' Company had given the hospital a bath chair during the year, for which thanks were expressed by the noble chairman. The building debt was cleared off in the year, and the income more than met the expenditure. The new year commences with a balance of £78 in the treasurer's hands.

---

The Blackheath Institute for Trained Nurses, 9, Montpelier Row, Blackheath, the lady superintendents of which are Mrs. L. Smith and Miss M. H. Duncan, is one of the few institutions which attempt to supply the want felt by that large section of the middle class who cannot afford the entire services of a trained nurse when they are ill. The Blackheath Institute, indeed, supplies nurses by the week; but increases where constant attention is not required their services can be had by the hour, morning or evening. They can thus go to the help of lonely invalids in lodgings, or to the relief of over-strained wives and mothers, who, however willing, could not afford to pay for their permanent attendance. This development of the nursing profession, in which we may flatter ourselves that our annotations on sick bachelors and their needs may have helped a little, promises to be one of its most useful and popular forms.

---

The people of Folkestone appear to be determined to quarrel with the local representatives of her Majesty's Government about the proposed site of the new hospital. The committee have selected as their site a piece of Government land. This seems to be the most suitable site obtainable, and was some time ago promised to them for £700. For some reason or other the price has been raised to £1,200. This, too, the committee are willing to give; but still delays are made. On Saturday evening an "indignation meeting" was held, at which the Mayor presided, and some strong language was iudulged in. The representatives of Government should endeavour to come to a decision, more especially as the proposed hospital is intended to be the Folkestone memorial of the Queen's Jubilee.

---

"C. P.," writing to the *Times*, says: "Will you allow me to supplement your leading article of yesterday upon lunatic asylums by pointing out a deficiency which has had little if any public notice? There are no lunatic asylums for the middle classes. The public asylums are for paupers, and no patient is permitted to pay more than what the pauper is reckoned to cost, which is, I believe, between £16 and £17 per annum, or to receive any exceptional food, treatment, or accommodation. On the other hand, all private asylums are for people of large means only, the payment ranging from £200 to £1,000 per annum. Now, the majority of lunatics, especially lunatic ladies, are perfectly able to feel the horror of being obliged to live in intimate association with persons of such language, manners, and habits as those who constitute a very large proportion of the inmates of every public asylum, and not only is confinement among such persons a hell upon earth to those who have been accustomed all their lives to the refinements of their class, but the chance of cure must manifestly be greatly diminished by so terrible a mental and moral irritation." Although "C. P." is right in assuming that special asylums for the middle classes should be provided, he is wrong in stating that there is no public asylum accommodation at present for such cases. Private asylums ought to be abolished, in the public interest, but meanwhile there are public asylums, like that at Morningside, Edinburgh, where private cases receive the best attention. Were it not for difficulties attaching to compensation, private asylums would have been closed in this country years ago, to the great benefit of the public and the patients, as well as that of the common weal.

